# Risk and Toxicity Assessment of a Potential Natural Insecticide, Methyl Benzoate, in Honey Bees (*Apis mellifera* L.)

**DOI:** 10.3390/insects10110382

**Published:** 2019-11-01

**Authors:** Yu-Cheng Zhu, Yanhua Wang, Maribel Portilla, Katherine Parys, Wenhong Li

**Affiliations:** 1 USDA-ARS-JWDSRC, Southern Insect Management Research Unit, Stoneville, MS 38776, USA; maribel.portilla@usda.gov (M.P.); katherine.parys@usda.gov (K.P.); 2 State Key Laboratory, Institute of Quality and Standard for Agro-Products, Zhejiang Academy of Agricultural Sciences, Hangzhou 310021, China; wangyanh79@hotmail.com; 3 Guizhou Academy of Agricultural Sciences, Guiyang 550006, China; yolanda_lwh@hotmail.com

**Keywords:** spray, contact, feeding, mixture, synergism, detoxification, P450, esterase, GST, bioassay

## Abstract

Methyl benzoate (MB) is a component of bee semiochemicals. Recent discovery of insecticidal activity of MB against insect pests provides a potential alternative to chemical insecticides. The aim of this study was to examine any potential adverse impact of MB on honey bees. By using two different methods, a spray for contact and feeding for oral toxicity, LC_50_s were 236.61 and 824.99 g a.i./L, respectively. The spray toxicity was 2002-fold and 173,163-fold lower than that of imidacloprid and abamectin. Piperonyl butoxide (PBO, inhibiting P450 oxidases [P450]) significantly synergized MB toxicity in honey bees, indicating P450s are the major MB-detoxification enzymes for bees. Assessing additive/synergistic interactions indicated that MB synergistically or additively aggravated the toxicity of all four insecticides (representing four different classes) in honey bees. Another adverse effect of MB in honey bees was the significant decrease of orientation and flight ability by approximately 53%. Other influences of MB included minor decrease of sucrose consumption, minor increase of P450 enzymatic activity, and little to no effect on esterase and glutathione S-transferase (GST) activities. By providing data from multiple experiments, we have substantially better understanding how important the P450s are in detoxifying MB in honey bees. MB could adversely affect feeding and flight in honey bees, and may interact with many conventional insecticides to aggravate toxicity to bees. However, MB is a relatively safe chemical to bees. Proper formulation and optimizing proportion of MB in mixtures may be achievable to enhance efficacy against pests and minimize adverse impact of MB on honey bees.

## 1. Introduction

Pollinators, including the honey bee (*Apis mellifera* L.), contribute $190 billion annually to global crop production [1]. In addition to the honey production, honey bees alone contributed 75% pollination and enhanced crop value annually by approximately $12 billion in the United States [1,2,3,4], being especially important to almond, apple, cherry, alfalfa, berries [3]. Unfortunately, losses of honey bee colonies due to some parasites and disease are not uncommon. The situation gained increased attention in late 2006 as some commercial beekeepers began reporting sharp declines in honey bee colonies. Due to the severity and unusual circumstances of these colony declines, scientists coined this phenomenon colony collapse disorder (CCD) [1]. Subsequent studies showed that CCD could be caused by tremendous pressure on honey bees from natural and human interferences, including parasites, pathogens, agrochemicals, and loss of natural forage [5,6,7].

The wide implementations of transgenic plants [8] have reduced the use of some insecticides, but have also caused a pest status shift from chewing insects to piercing/sucking insects on row crops [9,10]. This shift in pest complex, coupled with the development of insecticide resistance in target insects [11,12], has resulted in increased use of insecticide-treated seeds and foliar insecticides to control them in row crops. More than forty insecticides are currently recommended by extension specialists in the United States for the chemical control of row crop insects [13,14,15]. Most pesticides are applied as foliar sprays by airplane or ground sprayers. To increase control efficacy and control spectrum against crop pests, tank-mixing is a common practice for farmers to spray two different insecticides together, the mixture may pose additive or synergistic toxicity to honey bees as well.

More recently, concerns arose regarding the toxicity to honey bees from airborne insecticide dust during planting of insecticide-coated seeds [16] and increased pesticide residues in pollen and nectar from seed treatment with systemic neonicotinoids [17]. Residues of over 150 pesticides were detected at various levels in wax, pollen, bees, and honey found in hives [18,19]. The possible relationships between honey bee colony losses and sublethal effects of pesticide residues have received considerable attention, and previously published data indicate that pesticide residues may pose adverse impacts [20,21] or very low to no risk at all [22,23] to honey bee populations. While significant research efforts have been placed on the impact of residue levels of pesticides on honeybees, a number of important issues may have received much less research attention, including aggravated toxicity from using insecticide mixtures and alternative bio-insecticide developments.

Methyl benzoate (MB) is an organic compound, which exists in the freshwater fern *Salvinia molesta* M. [24]. With its pleasant smell, MB can be used to attract insects such as orchid bees, including *Euglossa cybelia* M. [25]. MB has also been isolated from honey bee volatiles that served as attractants to many lepidopteran species [26,27]. In addition, honey bee semiochemicals are attractive to small hive beetle (*Aethina tumida* M.), and the attraction was mediated by a blend of components dominated by an alarm pheromone [28], which includes isopentyl acetate (IPA), 2-heptanone, and methyl benzoate. Park et al. [29] reported that MB had fumigant activity against adzuki bean weevil (*Callosobruchus chinensis* L.) adults. An additional recent study found that MB exhibited acute insecticidal activity against various stages of several insect pests, including the brown marmorated stinkbug (*Halyomorpha halys* S.), diamondback moth (*Plutella xylostella* L.), tobacco hornworm (*Manduca sexta* L.)*,* and spotted wing drosophila (*Drosophila suzukii* M.) [30]. Additional laboratory toxicity data revealed that MB was at least 5 to 20 times more toxic than the conventional mixture of pyrethroid (*β*-cyfluthrin), sulfur, and pyrethrin [30]. Further work confirmed MB as a potential bio-insecticide against the red imported fire ant (*Solenopsis invicta* B.) [31] and silverleaf whitefly (*Bemisia tabaci* G.) [32].

Although MB has insecticidal activity against a wide variety of insect pests, it is important to understand whether MB is safe for application near both honey bees and other pollinators, as well as other beneficial insects. These other insects often play a valuable role in enhancing crop production. Other consideration is whether MB may adversely impact on honey bee physiology through interactions with other chemicals or other conventional insecticides to produce aggravated toxicity. The aim of this study is to examine the selectivity of MB and to minimize potential risk to honey bees and other pollinators while optimizing control efficacy against crop pests.

## 2. Methods and Materials

### 2.1. Honey Bee Hives

Honey bee colonies were originally purchased from commercial bee keepers located in pine forest and pasture area near Perkinston and Magee, Mississippi (USA). These colonies were maintained prior to research in a managed bee yard inside the Mississippi Wildlife Management Area located approximately 5 km north Stoneville (Mississippi, USA). Over the course of the research, approximately 15% new hives were purchased and established annually using package bees with Italian queens from commercial beekeepers near Little Rock, AR and added to the bee yard. Each hive was equipped with a bottom board oil trap (35 × 45 cm tray filled with vegetable oil) for monitoring and control of Varroa mite (*Varroa destructor* A.T.) and small hive beetle (*A. tumida*). To obtain bees (approximately 32,000) for conducting experiments, eight deep frames with more than 50% coverage of healthy sealed brood were pulled from 5–7 colonies and transferred into a vented container covered with a mesh screen and a lid to prevent bees from escaping. The container was held in a laboratory incubator (33 ± 0.5 °C; 60% ± 3 RH; no light). Each day, 20–25 newly emerged worker bees were transferred into a cage made with a 500-ml round wide-mouth polypropylene jar [D × H: 9.3 × 10 cm]) with a piece of plastic foundation (3 × 8 cm) attached vertically to the inner side of the cage. Each of these cages had an 8.9 cm diameter (d) hole cut in the lid and covered with 3 × 3 mm-mesh metal screen to prevent escape. Caged bees were provided with one scintillation vial (20 ml) of 50% (W/V) sucrose solution and one vial (20 ml) of water placed upside down on the top of each cage. Two holes (1.6 mm) were drilled in each vial cap. Caged bees were maintained in incubators (the same conditions described in 2.1) for 6–9 days before testing. Cages with more than four dead bees after 6–9 days (generally zero or one dead) were not used for experimentation. Before the experiment was started, dead bees were counted and excluded from the total number of bees tested in each replicate.

### 2.2. Chemicals

Methyl benzoate (MB), Tween 20, and Tween 80 were purchased from Fisher Scientific (Waltham, MA, USA). Piperonyl butoxide (PBO), triphenyl phosphate (TPP), and diethyl maleate (DEM) were purchased from Sigma-Aldrich (St. Louis, MO, USA).

Formulated insecticides, imidacloprid (Advise^®^ 2FL), acephate (Bracket^®^97), λ-cyhalothrin (Karate^®^), and oxamyl (Vydate^®^), were purchased from local agricultural chemical suppliers and kept in a refrigerator (6 ± 1 °C). These representative insecticides were used for preparations of binary mixtures with MB. Insecticide name, manufacturer, percentage of active ingredient (a.i.), spray treatment concentrations, field use (spray) concentrations of formulation, and mode of actions were listed in Table 1.

### 2.3. Bioassay Methods

#### 2.3.1. Dose Response Bioassay on Honey Bees 

A stock solution of methyl benzoate (MB) was prepared by mixing equal volume of MB and mineral oil. Appropriate volume of MB stock and d-H_2_O was transferred to new tube with additions of Tween 20 and Tween 80 at 1% each in final concentration [30] and vigorous shaking before being used for treatments.

To obtain contact median lethal concentration (LC_50_) of MB, serial dilutions of MB were made into d-H_2_O to create solutions in six concentrations: 5%, 8%, 12%, 18%, 27%, and 40%. A water (d-H_2_O) only and a mixture of Tween 20 and Tween 80 (each at 1%) were included as control. Each replicate consisted of one cage of 20 bees, all of which were seven d old, and three replicates were used for each treatment (either a concentration or control), for a total number of 24 cages of bees. To evaluate contact toxicity, a modified Potter Spray Tower was used to deliver 500 µl of the MB solution or d-H_2_O as control into each cage (containing 20 bees). The sprayer was set at 10 psi with spray distance of 22 cm, customized to ensure a uniform deposition of chemical mist on inner surface of the container and bees [34]. After spraying, caged bees were maintained in an incubator at the same conditions as described in 2.1. Mortality was recorded 48 h after treatment.

For testing oral toxicity to bees, appropriate volumes of MB and Tween 20/80 were well mixed with sucrose solution to make final volume of 20 ml in a standard scintillation vial. Six MB concentrations (same as in contact toxicity test) were prepared for the dose response assay. One vial of d-H_2_O and one vial of sucrose solution (containing MB and Tween 20/80) were placed upside down on the top of each cage which held 20 worker bees. Similar to the spray bioassay, each cage was considered a replicate, and three replicates were used for each of the six MB concentrations in addition to a non-MB control. Treated bees were maintained in an incubator at the same conditions as described in 2.1. Mortality was recorded 48 h after treatment. By using an analytic balance, the remaining MB + sucrose solution was measured for calculating 48-h consumption rate in bees treated with different concentrations of MB.

#### 2.3.2. Determine Major Enzymes for Detoxifying MB 

To test whether efficacy of MB was influenced by three major detoxification enzymes, three specific enzyme inhibitors were applied individually and in combination with MB. Cages of bees for this experiment contained 25 bees each in cages similar to above in 2.1, and all bees were nine days old. Each cage of bees was sprayed with 500 µl of PBO (piperonyl butoxide inhibiting cytochrome monooxygenase [P450]), TPP (triphenyl phosphate inhibiting esterase [EST)), or DEM (diethyl maleate inhibiting glutathione-S-transferase [GST]) solution at 1% (in 50% acetone) 1 h before spraying 15% MB solution. In addition to the enzyme inhibitors, a control containing water-only, 50% acetone, and 1% Tween 20/80 were also included. Five replicates, each consisting of a cage containing 25 bees, were used for each treatment and control. Post treatment or control application, bees were provided the same food source and incubated at the same environmental conditions described in 2.1. Dead bees were recorded, and mortality was determined after 48 h.

To further define the interaction of two chemicals (MB + inhibitor), additive (the effect of the mixture is equal to the sum of the individual effects), synergistic (greater than the sum of the individual effects), and antagonistic (less than additive) effects of the mixtures were determined according to the descriptions of Fernández-Alba et al. [35] and Preston et al. [36]. In this study we expanded additive effect of individual chemical A and B to a range between the greater than A or B effect and the A + B (sum) effect (A + B ≥ additive >A or B), because exact numerical A+B as additive effect may not exist. The effect less than the larger of A or B is antagonistic. In addition, effects were also subjected to statistical analysis (*p* = 0.05) to measure type of interaction involved (please see 2.5 for data processing and statistical analysis).

#### 2.3.3. Pesticide Interactions 

To test whether MB was able to interact with conventional chemical insecticides, binary mixtures were prepared by mixing MB solution (15% in final mixtures = LC_30_) with imidacloprid (Advise^®^ at 357 mg/L), acephate (Bracket^®^ at 103 mg/L), lambda-cyhalothrin (Karate^®^ at 362 mg/L), and oxamyl (Vydate^®^ at 180 mg/L). Individual chemical treatments at the same concentrations, water-only control, and 1% Tween 20/80 were also included. The final concentrations of MB were 15%, used for spray treatment to honey bees. The manufacturer, active ingredient (a.i.), and mode of action of four representative insecticides were shown in Table 1. Caged bees (25 bees/cage at age of nine days) were sprayed with 500 µl individual chemical or mixture solutions. Three cages were used as three replicates for each treatment. After treatment, bees were provided the same food source and incubated at the same environmental conditions described in 2.1. Mortality was recorded after 48 h. The interaction between MB and individual insecticides was defined based on the criterions of additive, synergistic, and antagonistic effects described in 2.3.2.

#### 2.3.4. Influence of MB on Honey Bee Feeding 

At the beginning and end of feeding dose response assay (described in 2.3.1.), the starting and remaining weight of sucrose solution was measured using the analytic balance. Sucrose consumption per bee was calculated by dividing the consumed volume by the average number of surviving bees between 0 (before treatment) and 48 h.

#### 2.3.5. Flight Test 

This experiment was designed to test whether bees were able to normally orient and achieve flight after experimental MB treatments. Preliminary data and experiments were conducted to optimize procedures for scoring the flight performance of bees after treatment (see descriptions in Table 2). Caged bees (20/cage) at seven d old were treated separately using contact or feeding methods. MB concentrations and control were the same as described in 2.3.1. After spray or feeding treatments for 48 h, three surviving bees were randomly collected as a replicate, and three replicates were used for each treatment. Surviving bees were tested individually for their ability to fly 3.66 meters (12 ft.) toward a window within a min. All lights were turned off and a window (145 × 117 cm) was left uncovered. Room temperature was set at 27 ± 1 °C. Treated bees were individually released into a 150 cm petri dish located 3.66 m from the window and a timer was set to run 1 min. The flight performance of each bee was judged by the following categorical standards described in Table 2.

### 2.4. Enzyme Activity Assays

Enzyme activities of P450, EST, and GST were assayed using corresponding substrates in surviving bees after contact or feeding bioassays with MB. MB was serially diluted to six concentrations (5%, 8%, 12%, 18%, 27%, and 40%), and caged bees were treated with both contact or feeding methods. After 48 h, surviving individuals were collected for enzyme activity assays.

In an additional experiment, caged bees were treated with enzyme inhibitors and MB at 15% (treatment methods described in 2.3.3). Enzyme preparations and enzyme activity quantifications were processed according to the procedures of Zhu et al. [37]. Relative enzyme activities were calculated as the ratio of the activity of MB or MB + inhibitor treatment to the activity of water-only control in surviving bees after spraying or feeding treatments for 48 h.

### 2.5. Data Processing and Statistical Analysis

SAS (version 9.4) [38] was used for probit analysis to calculate LC_50_ values of MB dose response (spray and feeding) assays, and Chi-square test was applied to ensure the goodness-of-fit of the model. Analysis of variance (ANOVA) and Proc GLM (general linear model) procedure was applied with the option of Fisher’s LSD (least significant difference) method for mean separation at *p* = 0.05. To confirm synergistic or additive interaction of MB with enzyme inhibitor or conventional insecticide, additional t-tests (Sigmaplot Analysis) were applied to reveal statistical difference between the combined mortality of two separate chemical treatments and the mortality of mixture of the same (two) chemicals.

## 3. Results

### 3.1. Spray and Feeding Toxicities of MB to Honey Bees 

Two separate dose response bioassays were conducted using spray or feeding methods in this study to reveal the toxicities of MB to honey bees (Table 3). LC_50_ values represent the chemical concentration to generate 50% mortality in a population of the test organism. Probit analysis showed that none of the parameters (toxicity, 95% Fiducial Limits, and slope) were overlapping each other between spray and feeding treatments. The slope of dose response curve via spray was 1.63-fold higher than the slope via feeding treatment, indicating honey bees were more sensitive to changes of spray concentrations. Therefore, the data indicated a significant difference between two different treatment methods. The ratio of contact toxicity via spraying to oral toxicity via feeding was 3.49-fold based on LC_50_. After each LC_50_ was converted to LD_50_ (Table 3), toxicity ratio of contact to oral toxicity was increased further to 107-fold.

### 3.2. Examination of Major Enzymes for Detoxifying MB 

**Figure 1:**
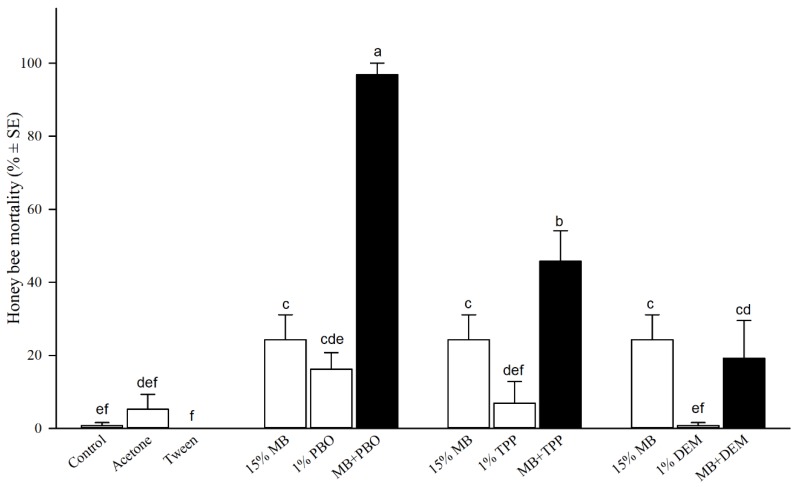
Examination of major enzymes on detoxifying methyl benzoate (MB) in honey bees using piperonyl butoxide (PBO: Inhibits P450 oxidase), triphenyl phosphate (TPP: Inhibits esterase), or diethyl maleate (DEM: Inhibits glutathione S-transferase). Mean bars with the same letters at the top of error bars indicate no significant difference between treatments (*p* > 0.05). The treatment of 15% MB was reused for side-by-side graphical comparison within each of three inhibitor groups.

When 1% PBO (piperonyl butoxide) and 15% MB were sprayed individually to honey bees, 48-h mortality was 16% and 24%, respectively (Figure 1). The mortality increased to 97% when bees were sprayed with both PBO + MB, significantly higher (*p* < 0.05) than both individual treatment and substantially higher than the sum (40%) of individual treatments of PBO and MB, indicating a synergistic interaction between PBO and MB (*F* = 29.35, df = 9, *p* < 0.0001). Additional t-test showed significantly higher mortality of mixture (MB+PBO) treatment than combined mortalities of separate MB and PBO treatments, therefore, confirmed the synergistic effect of MB with PBO (t = 8.624, *p* < 0.0001). Spray treatment of 1% TPP (triphenyl phosphate) plus 15% MB increased honey bee mortality to 46%, significantly higher than either TPP (7%) or MB (24%) applied individually (Figure 1). However, additional t-test did not confirm synergistic interaction of between MB and TPP (t = 1.035, *p* > 0.05), and the interaction between MB and TPP was characterized as additive. DEM (diethyl maleate) plus MB treatment produced 19% bee mortality, lower (though not significantly different in both mean separation and t-test, *p* > 0.05) than MB only, indicating no interaction between MB and DEM.

### 3.3. Interaction of MB with Four Conventional Insecticides 

**Figure 2:**
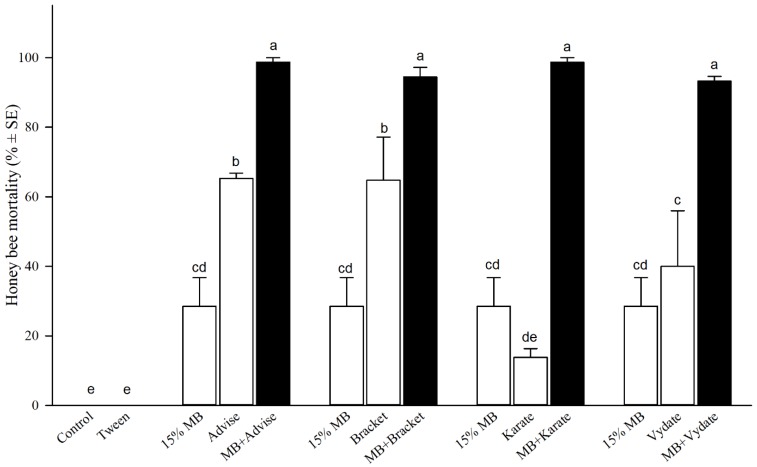
Synergistic spray toxicity of methyl benzoate (MB at 15%) mixed with four representative insecticides (classes), imidacloprid (Advise^®^ at 357 mg/L), acephate (Bracket^®^ at 103 mg/L), lambda-cyhalothrin (Karate^®^ at 362 mg/L), and oxamyl (Vydate^®^ at 180 mg/L) in honey bees. Mean bars with same letters at the top of error bars indicate no significant difference between treatments (*p* > 0.05). The treatment of 15% MB was reused for side-by-side graphical comparison within each of four insecticide groups.

All binary mixtures of 15% MB with each of four insecticides (Advise^®^ 2FL [imidacloprid], Bracket 97^®^ [acephate], Karate^®^ [λ-cyhalothrin], and Vydate^®^ [oxamyl] representing four classes) showed significantly higher mortality than individual treatment (Figure 2; [*F* = 34.48, df = 10, *p* < 0.0001]). Bioassays using the spray tower revealed that 48-h bee mortalities of individual MB and Advise were 29% and 65%, respectively (Figure 2). The binary mixture of MB + Advise increased bee mortality to 99%, significantly higher than (*p* < 0.05) both individual treatments. Since the mortality from MB + Advise treatment was greater than the sum of the individual mortalities of MB and Advise treatments, synergistic interaction might exist between MB and Advise. Similarly, the rest of three binary mixtures (Figure 2) also showed higher bee mortalities than those corresponding individual insecticide treatments, indicating potential synergistic interaction between MB and these four representative insecticides. However, additional t-tests confirmed a synergistic interaction only between MB and Karate^®^ [λ-cyhalothrin] with significantly higher mortality from mixture (MB + Karate) treatment than combined mortality of separate MB and Karate treatments (t = 7.897, *p* < 0.05). Other three conventional insecticides showed additive (not synergistic, *p* > 0.05) interactions with MB after t-tests.

### 3.4. Influence of MB on Honey Bee Feeding 

**Figure 3:**
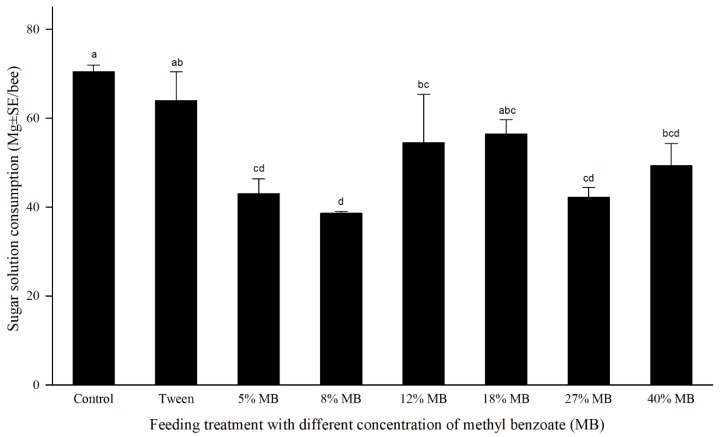
Influence of methyl benzoate (MB) on honey bee feeding. Sugar solution consumptions were calculated based on number of live bees averaged between 0 and 48 h after treatment. Mean bars with the same letters at the top of error bars indicate no significant difference between treatments (*p* > 0.05).

Results of MB influence on sucrose solution consumption in honey bees are shown in Figure 3. The effect of treatments substantially influenced the feeding of sucrose solution in honey bees, but the overall difference was not significant (*F* = 4.66, df = 7, *p* =0.0051). All MB treatments at six different concentrations showed relatively lower sucrose ingestion than control, but not all concentrations showed statistical difference and the reduction was not correlated closely with MB concentrations. The treatment with 1% Tween 20/80 (1:1) reduced honey bee feeding by approximately 9.2%, but the difference was not statistically significant from water-only control. A half of MB concentrations showed similar sucrose solution ingestion and the other half MB treatments had significantly lower consumption rates than the Tween-only treatment although all MB treatments contained the same concentration of Tween (Figure 3).

### 3.5. Impact of MB on Honey Bee Flight 

**Figure 4:**
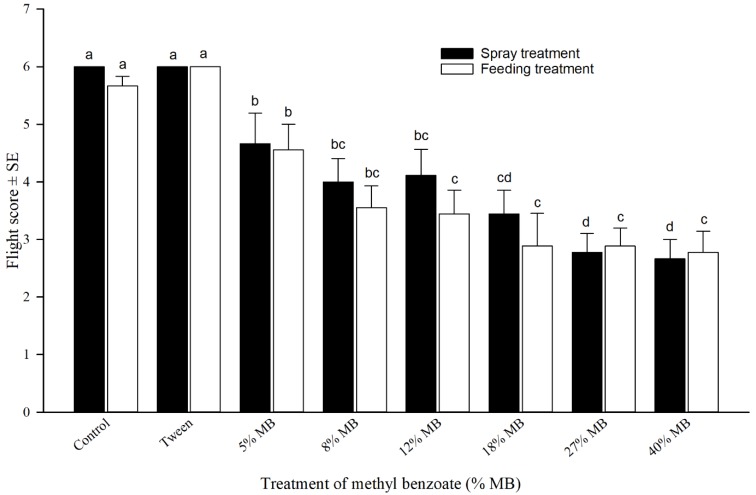
Influence of methyl benzoate (MB) on honey bee flight (performance score for flying 3.66 m). Mean bars with the same letters at the top of error bars indicate no significant difference between treatments (*p* > 0.05).

**Figure 5:**
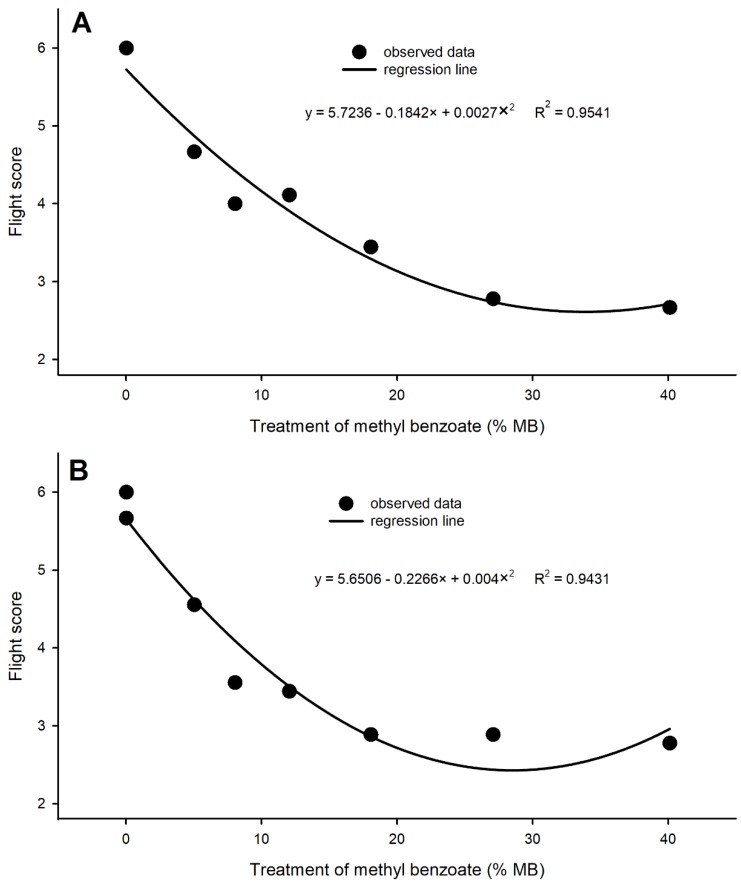
Regression analysis of flight score against methyl benzoate concentrations in honey bees treated with spraying (**A**) and feeding (**B**) method.

Treated bees either by spraying or feeding 1% Tween 20/80 (1:1) showed no difference in flight score from water-only control, indicating no visually observed negative impact from Tween on bee flight (Figure 4). Bees were able to orient toward the light from the window, could hover or fly steadily, and reached the window within 30 s. Even at the lowest concentration tested (5%), MB significantly reduced the flight ability of honey bees. The influence on flight score seemed negatively correlated to the concentrations of MB. A quadratic relationship between flight scores and MB concentrations was established (Figure 5, using the dynamic fit regression in Sigmaplot V13). After spray treatment, honey bee flight scores decreased as MB concentrations increased followed the model y = 5.7236 − 0.1842× + 0.0027×^2^ with R^2^ = 0.9541 (Figure 5A). Bees fed on MB containing sucrose solutions showed gradually decreased flight scores as MB concentrations increased in a trend of y = 5.6506 − 0.2266× + 0.004×^2^ with R^2^ equal to 0.9431 (Figure 5B).

### 3.6. Effect of MB on Honey Bee Detoxification Systems

**Figure 6:**
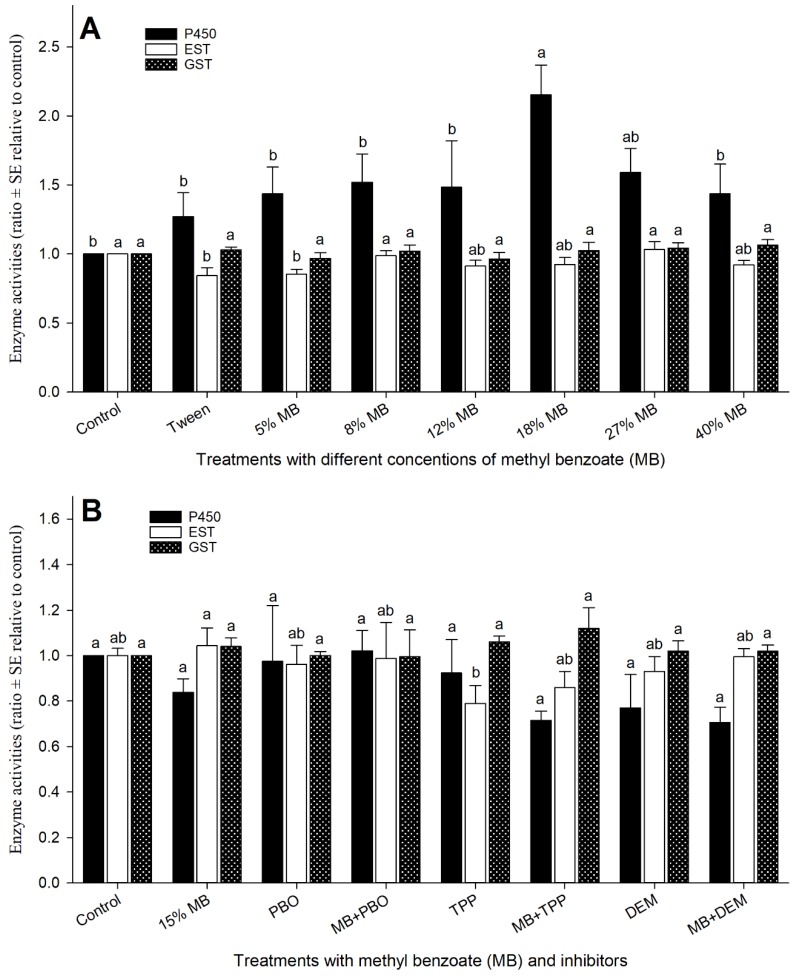
Impact of methyl benzoate (MB) on three detoxification enzymes in honey bee survivals after treatments with six different concentrations of MB (**A**) and detoxification enzyme inhibitors (**B**). Mean bars with the same letters at the top of error bars indicate no significant difference between treatments (*p* > 0.05) within enzyme group. PBO: Piperonyl butoxide which inhibits P450 oxidase (P450); TPP: Triphenyl phosphate which inhibits esterase (EST); and DEM: Diethyl maleate which inhibits glutathione S-transferase (GST).

Enzyme activities of cytochrome P450 oxidases (P450s), esterases (ESTs), and glutathione-s-transferases (GSTs) were examined in bees that survived the bioassays. Among the three major enzymes examined, the P450 activity showed the greatest variations in response to the changes of MB concentrations (Figure 6A). It seemed that P450 activity increased as MB concentrations increased until the activity reached a peak (2.15-fold) after MB concentration increased to 18%. The activity fell down as MB concentration increased to 27% and 40%. However, all treatments (MB concentrations) had higher P40 activity than water-only control. Esterase activity of 5% MB was lower than that of the control, but all other MB concentrations had no influence on EST activity. Similarly, all MB concentrations (ranging from 5% to 40%) consistently exhibited similar GST activities indicating MB had no effect on GSTs (Figure 6A). By applying enzyme inhibitors and MB individually or in combination (Figure 6B), P450s activities were seemly lower than control, but no statistical differences were observed. As expected, TPP significantly reduced EST activity, while all other treatments did not alter GST activity significantly. Again, all individual and combined treatments of MB and inhibitors did not alter GST activities in honey bees (Figure 6B).

## 4. Discussion

Common insect pests found in southern row crops share a broad range of ecosystems with pollinators and other beneficial insects. Improper usage of insecticides for controlling pest populations may accidently cause poisoning of honey bees. Discovery and development of novel insecticides from natural products may have a bright future in pest control if selective agents can be found. Methyl benzoate (MB), an ester, was isolated from honey bee volatiles serving as attractants [26,27,28]. Insecticidal activity of MB was confirmed by Feng and Zhang [30]. The present study was extensively focused on risk assessments of MB on honey bees. By using feeding methods to simulate in-hive exposure and a spraying method to simulate in-field exposure, this study makes significant contributions for better understanding (1) contact and oral toxicities of MB to honey bees; (2) major enzymes for detoxifying MB; (3) interaction with conventional insecticides representing four commonly used insecticide classes; (4) influence on feeding in honey bees; (5) impact of MB on honey bee flight; and (6) effect of MB on detoxification enzyme activities.

Median lethal concentrations (LC_50_) were obtained via spray, which is the most commonly utilized method for pest control in row crop agriculture. To achieve 50% mortality in honey bee workers, a spray concentration containing 22.13% MB or 236.61 g pure MB per liter was required. By comparing this number with the LC_50_ (mg a.i./L) of 42 pesticides commonly recommended by extension agents for southern row crop pest control [34], we are able to rank the toxicity of MB on honey bees. Results indicated that the toxicity of MB on honeybees was ranked 35th of the 43 chemicals (Table S1), lower than all conventional insecticides. Abamectin is the most toxic insecticide to honey bees among the 42 previously tested pesticides, which is 173, 163-fold higher toxic to honey bees than MB. Imidacloprid, acephate, λ-cyhalothrin, and oxamyl represent four of the major classes of insecticides (neonicotinoids, organophosphates, pyrethroids, and carbamates, respectively) and were observed to have 2,002-, 1,929-, 1,804-, and 2,632-fold higher toxicity than MB to honey bees. MB is relatively safe to honey bees based on its toxicity from contact with the chemical. When comparing toxicity of a chemical using different exposure routes, most insecticides (including neonicotinoids, organophosphates, and carbamates) have higher oral toxicity than contact toxicity, with a major exception being pyrethroids that have higher contact toxicity in honey bees. In this study, MB (both LC_50_ and LD_50_ in Table 3) showed substantially higher contact toxicity than oral toxicity, indicating that toxicity from MB acts mainly through cuticular penetration. However, feeding reduction (approximately 0.24 to 0.82-fold decrease) may compromise certain feeding toxicity. Regarding MB toxicity to beneficial pollinator compared to crop pest, we currently expand assessment of MB toxicity against an economically important crop pest, the tarnished plant bug (*Lygus lineolaris* B.). Our preliminary data indicated that MB had a several-fold higher toxicity to tarnished plant bugs than to honey bees, seemly an advantage of selectivity against target pests (Data not shown). Mostafiz et al. [32] also used a spray method to treat adult *B. tabaci* and found MB was very effective (LC_50_ = 0.2%) against the insect. Comparisons between methodologies utilized by Mostafiz et al. [32] and our present study indicated that their container for holding flies had a substantially smaller hole than ours, which may effectively prevent MB from vaporizing. Nonetheless, our treatment method is designed to mimic in-field spraying conditions. While MB is volatile, this vaporization may substantially reduce its insecticidal potential in some systems. A stable formulation would need to be developed and optimized to enhance its insecticidal activity against crop pests and minimize direct contact to beneficial insects.

To determine how MB is detoxified in honey bees, we examined the influence of three major detoxification enzymes: Cytochrome P450 oxidases (P450s), esterases (ESTs), and glutathione S-transferases (GSTs) on MB by applying three specific enzyme inhibitors in conjunction with MB to honey bees. By applying PBO (inhibits P450s), TPP (inhibits ESTs), or DEM (inhibits GSTs) individually or in combination with MB to honey bees, a significant increase in mortality in MB + treatment over the mortality observed from either the two individual treatments alone was an indication of interaction of the two chemicals, additively or synergistically. In the case of MB+PBO (Figure 1), MB and PBO each applied to honey bees alone caused less than 28% mortality, but MB+PBO produced more than 2.4-fold higher (97%) mortality than the sum effects of two individual chemicals in honey bees. The synergistic interaction could be incurred by direct suppression of P450s jointly by MB and PBO or as an indirect effect of MB on the insect’s physiology to make test subjects weaker or more susceptible to the application of PBO. TPP (esterase inhibitor) incurred additive toxicity, but DEM (GST inhibitor) showed no interaction with MB in bees. A significantly higher increase of bee mortality rate by PBO than by TPP indicating that P450 oxidases are major and dominant detoxification enzymes, while esterases have certain but GSTs have no effect on MB detoxification in honey bees.

As formulated premixtures and tank mixing are popular practices in commercial pesticide application found in grower fields, we selected representative insecticides from four different common classes of insecticides to test binary mixtures in conjunction with MB. Different classes of insecticides have different modes of action (MOD) designed to reach specific target sites. A popular premixture Endigo formulated by Syngenta contains the chemical thiamethoxam, which exhibits systemic activity to target insects with piercing-sucking mouthparts, through competitive binding to nicotinic acetylcholine receptors (nAChR) and λ-cyhalothrin with a fast contact toxicity for chewing insect control through sodium channel modulation [33]. It is desirable to use mixtures of insecticides to increase both pest control efficacy and the spectrum of control. These studies revealed potential aggravated toxicity against honey bees from potential mixtures and to provide further information for selecting insecticides that could cause aggravated toxicity to pollinators. Unfortunately, all four mixtures of MB with the representative insecticide classes incurred significant additive toxicities to honey bees with synergistic interaction detected in the mixture of MB with λ-cyhalothrin. While the study only examined one representative from each of the four insecticide classes, it is possible that different chemicals within a class of insecticide will exhibit differing levels of interactions with MB. In addition, modifying the proportions of each component within a mixture could help to develop better mixtures for minimizing additive and/or synergistic toxicities to honey bees as well as producing better efficacy against a broad range of insect pests.

Experimental honey bees treated with MB (containing 1% Tween) consumed approximately 26%–45% less sucrose solution containing six different concentrations of MB (ranging from 5%–40%). The tween-only control also exhibited certain reduction in feeding, although the difference was not statistically different from water-only control. There was no trend between level of feeding inhibition and concentration of MB. Reduced feeding was also observed in bees after treatment with insecticides [40,41].

Influence of chemicals on honey bee flight is an important parameter for assessing negative impact of pesticides to worker bees. Studies have shown that chronic exposure to pesticide residues has adverse impacts on bees, including impaired immune function, learning, orientation, foraging, and motor coordination [42,43]. Other studies have previously demonstrated changes in olfaction, learning, and orientation in honey bees upon exposure to neonicotinoids. Exposure to levels of imidacloprid above 20 μg/kg can lead to both physiological and behavioral abnormalities in honey bees, including decreases in learning, fecundity in queens, and foraging activity, in addition to the potential of increased susceptibility to other environmental stressors [44,45,46,47]. Radiofrequency identification (RFID) techniques have been previously used to examine the influences of insecticides on honey bee foraging and homing activities by gluing a tiny chip on the back of a limited number of bees from each hive [48,49]. However, a feasible and robust testing technique has not been developed and widely used to assess large number of free-flying bees after treatment of insecticides. To quantify honey bee orientation and flight performance, we developed a method and established criteria for scoring honey bee flight ability after chemical treatment. This method was refined after several years of preliminary experiments. In this study, we used a room with a window that provided natural light to induce honey bees to fly a fixed distance of 3.66 m after treatment. With an easy scoring standard, we quantified and obtained consistent flight data following both spray and feeding treatments. Results indicated that MB adversely impaired both orientation and flight. The influence was negatively correlated with MB concentrations in a quadratic relationship (Figure 5). With this technique available, we are able to more easily understand how chemical insecticides impact on honey bees.

In vitro activities of cytochrome P450 oxidases (P450s), esterases (EST), and glutathione S-transferases (GSTs) were also monitored in honey bees using corresponding substrates. Most P450s perform a variety of functions by catalyzing the oxidation of organic substances to fulfill many tasks, including the synthesis, degradation, and metabolic intermediations of lipids, ecdysteroids and juvenile hormones, and metabolizing of substances from a variety of origins [50]. ESTs in insects are frequently implicated in the detoxification or resistance to synthetic insecticides in the organophosphate, carbamate, and pyrethroid classes, mainly through altering gene amplification and upregulation [51]. GSTs catalyze the secondary metabolism of a wide variety of compounds oxidized by P450s [52]. These catalysis reactions transform a wide range of internal and external compounds, including herbicides and insecticides [53]. Examinations of these three detoxification enzymes in this study demonstrated that EST and GST activities were consistently similar to the results seen in the water-only control. The insensitivity of EST and GST to treatments may be explained by a variety of reasons including: EST and GST activities were obtained from surviving bees that may have physiologically recovered from treatment after 48 h; the activity of EST or GST was detected as total enzyme activity, and some of those enzymes may not function for chemical detoxification; honey bees lack genetic diversity with as few as half of the detoxification genes of either *Drosophila melanogaster* or *Anopheles gambiae* G. [54,55,56]. Iwasa et al. [57] suggested that ESTs and GSTs appeared to be less important in honey bee detoxification systems based on limited data from honey bees. Synergizing MB toxicity in this study and imidacloprid toxicity [58] provided evidence that P450 oxidases are major detoxification enzymes in honey bees. Our in vitro enzymatic data also partially supports that the influence of MB to bees may be mediated by P450 oxidases. Lacking consistent P450 activity data for all experiments could be explained by the same reasons for EST and GST (above). Considering that different P450 oxidase may play different roles in metabolic pathways, future work will examine which P450 gene expression is upregulated and which is downregulated to shed light on the involvement of each P450 gene in MB and other insecticides detoxifications.

## Figures and Tables

**Table 1:** Pesticide name, manufacturer, percentage of active ingredient, spray concentration of formulation, and mode of action.

Chemical Name (Active Ingredient)	Commercial Name	Manufacturers	Active Ingredient a.i.%	Spray Concentration of Formulation (mg/L)	Mode of Action [33]
Imidacloprid	Advise 2FL	Winfield Solutions LLC	21.4	357	Nicotinic acetylcholine receptor (nAChR) competitive modulators
Acephate	Bracket97	Winfield Solutions LLC	97	103	Acetylcholinesterase (AChE) inhibitors
l-Cyhalothrin	Karate Z	Syngenta	22.8	362	Sodium channel modulators
Oxamyl	Vydate	DuPont	42	180	Acetylcholinesterase (AChE) inhibitors

**Table 2:** Standards for scoring honey bee orientation and flight performance after chemical treatment.

Score	Descriptions of Flight Performance
0	The bee was motionless, dead, or unable to walk for a whole min
1	The bee walked or flapped wings, but stayed within the petri dish for the whole min
2	The bee flew out the petri dish, fell to the floor, and stayed on the floor most of the time with occasional taking off and falling to floor
3	The bee flew and landed randomly without orientation toward sunlight (window) for the whole min
4	The bee flew toward sunlight quickly and hit the window in less than 3 s (but the flight was not normally performed by hovering or flying steadily toward sunlight to reach window in 6–15 s)
5	The bee took 31–60 s to fly the distance toward sunlight and landed on the window, including stopping, turning, and flying backward
6	The bee flew steadily and directly toward sunlight and landed on window in 4–30 s (most took 6–15 s with zigzagging left and right in a range of 30 cm without stopping, turning, or flying backward)

**Table 3:** Spray and feeding toxicity (48 h) of methyl benzoate to honey bee workers.

Treatment Method	Toxicity (LC_50_ [g a.i./L])*	95% Fiducial Limits	Pr > ChiSq	Slope±SE^b^	Predicted LD_50_ (mg a.i/bee)*
Spray	236.61	210.74–269.76	0.9447	1.3669 ± 0.1357	0.37**
Feeding	824.99	488.84–2905.55	0.3139	0.519 ± 0.1176	39.60***

* Toxicity (LC_50_ g [a.i.]/L) was calculated based on 99% of MB active ingredient (a.i.) and weight of 1.08 g/ml. ** LD_50_ mg/bee was calculated by multiplying LC_50_ value by the volume of MB solution deposited on each worker bee (1.575 µl/bee) reported by Zhu et al. [34]. *** LD_50_ mg/bee was calculated by multiplying LC_50_ value by 48 µl of MB-sucrose solution consumed in two days by each worker bee (daily consumption of 24 µl/bee was reported by Free and Spencer-Booth [39].

## Supplementary Materials

The following are available online at https://www.mdpi.com/2075-4450/10/11/382/s1, Table S1: Comparison of spray toxicity of methyl benzoate (MB) to 42 conventional pesticides recommended for field crop pest control.
