# Supplementary material for: Risk and Toxicity Assessment of a Potential Natural Insecticide, Methyl Benzoate, in Honey Bees (Apis mellifera L.)
Source: Insects. 2019 Nov 1;10(11):382. doi: 10.3390/insects10110382 (PMC6920933; doi:10.3390/insects10110382)
Supplement: Supplementary file 1 [file insects-10-00382-s001.pdf]

Sup_Table S1. Comparison of spray toxicity of methyl benzoate (MB) to 42 conventional pesticides recommended for field crop pest control [34]

Chemical Name	LC_{50} mg (a.i.)/L	Toxicity Rank by LC_{50}	Relative toxicity to MB
Abamectin	1.37	1	173163
Emamectin Benzoate	1.41	2	167991
Zeta-cypermethrin	13.28	3	17820
Thiamethoxam+l-cyhalothrin	13.52	4	17498
Clothianidin	15.88	5	14904
Bifenthrin+avermectin	17.43	6	13572
Dicrotophos	20.43	7	11579
Thiamethoxam	25.02	8	9455
Beta-cyfluthrin	39.64	9	5969
Imidacloprid+b-cyfluthrin	44.76	10	5287
Cyfluthrin	45.16	11	5239
Methomyl	52.02	12	4548
Bifenthrin+Zeta-cypermethrin	56.06	13	4221
Dimethoate	62.11	14	3810
Bifenthrin	64.83	15	3650
Chlorpyrifos	67.73	16	3494
Permethrin	72.96	17	3243
Gamma-Cyhalothrin	82.84	18	2856
Oxamyl	89.89	19	2632
Cypermethrin	101.76	20	2325
Sulfoxaflor	114.75	21	2062
Imidacloprid	118.17	22	2002
Acephate	122.64	23	1929
Esfenvalerate	126.00	24	1878
Lambda-Cyhalothrin	131.19	25	1804
Spinosad	133.60	26	1771
Indoxacarb	171.00	27	1384
l-cyhalothrin+chlorantraniliprole	190.66	28	1241
Methoxyfenozide+spinetoram	201.56	29	1174
Carbaryl	394.79	30	599
Thiodicarb	603.16	31	392
Fenpyroximate	732.25	32	323
Tetraconazole	1661.53	33	142
Acetamiprid	1.76E+05	34	1
MB (methyl benzoate)	236610	35	1
Flonicamid	4.88E+05	36	0.48481
Etoxazole	9.33E+05	37	0.25347
Novaluron	3.75E+06	38	0.06308
Propargite	5.61E+07	39	0.00422
Flubendiamide	2.32E+08	40	0.00102
Chlorantraniliprole	1.46E+16	41	0.00000
Spiromesifen	6.36E+18	42	0.00000
Glyphosate	2.25E+34	43	0.00000
